# Risk stratification for postoperative hematoma following anterior cervical spine surgery: a machine learning approach

**DOI:** 10.1016/j.xnsj.2026.100909

**Published:** 2026-06-01

**Authors:** Taha M. Taka, Andrew Cabrera, Alexander Bouterse, Henry Avetisian, David Shin, Udochukwu E. Oyoyo, Olumide Danisa

**Affiliations:** a Department of Orthopedic Surgery, Loma Linda University Health, 11234 Anderson Street, Loma Linda, CA 92354, United States; b Department of Orthopedic Surgery, Tripler Army Medical Center, 1 Jarrett White Road, Honolulu, HI 96859, United States; c Jacobs School of Medicine, University at Buffalo, 955 Main Street, Buffalo, NY 14203, United States; d School of Medicine, Loma Linda University Health, 11175 Campus Street, Loma Linda, CA 92350, United States; e Dental Education Services, Loma Linda University School of Dentistry, 11092 Anderson Street, Loma Linda, CA 92350, United States; f Departments of Orthopedic Surgery and Neurological Surgery, Duke University Health System, 200 Trent Drive, Durham, NC 27710, United States

**Keywords:** Cervical, Hematoma, Machine learning, ACDF, Spine, Propensity score matching

## Abstract

**Background:**

Anterior approaches to the cervical spine have consistently increased annually, with commonly performed procedures demonstrating low morbidity and mortality rates. However, rare complications of postoperative hematoma requiring readmission or reoperation poses risks such as respiratory compromise and reintubation. This study sought to utilize machine learning algorithms (MLA) on American College of Surgeons National Surgical Quality Improvement Program data to characterize clinical risk profile for readmission and reoperation secondary to postoperative hematoma in patients following anterior cervical procedures.

**Methods:**

A query of the American College of Surgeons National Surgical Quality Improvement Program database identified adult patients undergoing elective anterior cervical spine procedures from 2012 to 2018 and who developed a postoperative hematoma requiring readmission or reoperation within 30 days. 1:5 Propensity score matching was employed to ensure comparable groups and reduce data bias. Six MLAs were constructed to evaluate the association of preoperative variables with postoperative hematoma development within the matched cohort. Permutation feature importance (PFI) was derived from the top-performing MLA to identify and quantify the relative contribution of individual clinical factors to overall risk variance.

**Results:**

Of 54,427 patients, following the 1:5 Propensity score matching, 1,056 patients remained, with 176 (16.67%) developing a postoperative hematoma that required either readmission and/or reoperation. The 6 MLAs generated predictions with an average AUC of 0.824 and average accuracy of 87.75%. However, reflecting the class imbalance of this exceedingly rare complication, the models demonstrated a low average sensitivity of 30.2%. Analysis of PFIs from the top performing algorithm identified diabetes (PFI = 0.020, p = .020), history of smoking (PFI = 0.026, p = .026), preoperative WBC (PFI = 0.028, p = .028), preoperative sodium (PFI = 0.001, p = .001), and dependent function status (PFI = 0.001, p = .001) as statistically significant preoperative factors in the development of postoperative hematomas.

**Conclusions:**

MLAs identified several variables associated with clinically significant postoperative hematoma formation following anterior cervical spine surgery. However, given the low sensitivity driven by the rarity of this event, these algorithms cannot reliably predict individual patient outcomes or serve as independent screening tools. Instead, they function as adjunctive assets to enhance perioperative risk awareness and guide risk stratification.

## Introduction

The anterior approach to the cervical spine has become widely employed in the treatment of cervical spine pathologies and is currently utilized in more than 80% of cervical fusions performed in the United States [1,2]. Anterior cervical discectomy and fusion (ACDF), cervical disc arthroplasty (CDA), and anterior cervical corpectomy (ACC) are among the most common procedures to apply this approach, accounting for more than 1.06 million primary operations between 2006 and 2013 and over 132,000 procedures performed annually [1]. While these surgeries have demonstrated markedly low rates of periprocedural morbidity and mortality, the complex vascular, neural, pulmonary, and digestive anatomy involved with each may pose significant challenges during surgical dissection. Most notably, postoperative hematoma, though extremely rare, is a potentially devastating sequelae of anterior approach surgeries that may necessitate unplanned readmission and reoperation to prevent respiratory compromise. As the prevalence of outpatient anterior cervical surgeries has grown, the need to identify key drivers of clinically significant postoperative hematoma has become increasingly relevant to the enhancement of perioperative planning, patient monitoring, and disposition [2,3]. Previous studies estimate the incidence of postoperative hematoma following anterior cervical surgeries to be relatively low, at 0.2% to 1.9%; however, due to its substantial risk profile, including an increased likelihood of postoperative reintubation, surgical site infections, and pulmonary complications, further investigation into the factors that predict this outcome may be warranted [4,5].

Machine learning (ML), an application of artificial intelligence, has been widely adopted as an effective means of large-scale data processing and predictive analysis within medical research [6,7]. Specifically, ML classification algorithms are capable of identifying and applying learned patterns within clinical datasets to generate efficient and accurate predictions of selected patient outcomes, including perioperative morbidity and mortality [[8], [9], [10], [11]]. As such, the application of this methodology to predict variables associated with postoperative hematoma may serve to identify clinically relevant risk factors for patients undergoing anterior-approach cervical procedures. Therefore, the purpose of this study was to employ 6 ML classifiers to characterize risk factors associated with readmission and reoperation in patients experiencing postoperative hematoma following anterior approach cervical procedures using the ACS-NSQIP database.

Prior investigations of clinically significant postoperative hematoma have relied predominantly on traditional multivariable regression techniques to identify independent risk factors [4,[12], [13], [14]]. While regression-based models are effective for hypothesis testing and estimation of linear associations, they are limited in their ability to capture nonlinear relationships and higher-order interactions among perioperative variables. Conversely, supervised ML approaches can accommodate complex feature interactions and may offer complementary insight into risk stratification for postoperative complications. However, it is important to note that given the low incidence of clinically significant postoperative hematoma in anterior cervical spine surgery, predictive modeling is inherently challenged by class imbalance and the resulting models are limited by low sensitivity. Therefore, these algorithms must be interpreted as adjunctive tools for exploratory risk stratification, aimed at increasing clinical vigilance rather than replacing standard clinical judgment. Accordingly, the novelty of this study lies not only in the development of new algorithms, but in the application and direct comparison of ML classifiers against a conventional logistic regression baseline to evaluate their relative performance and feature importance in this clinical context.

## Materials and methods

After receiving exemption from our institution’s review board (IRB# 5240030), the ACS-NSQIP database was queried using RStudio (RStudio, PBC) to identify adult patients aged 18 to 85 years old undergoing elective anterior cervical spine surgery from 2012 to 2018 using the following current procedural terminology codes: 22551 and 22552 for ACDF, 22554 and 22585 for ACD without fusion, 22856 and 22858 for cervical disc displacement, and 63081 and 63082 for corpectomy. Patients who required readmission and/or reoperation due to postoperative hematoma formation were identified using International Classification of Diseases (ICD-9) codes 998.11, 998.12, and 998.13 and ICD - 10 codes G97.5, G97.51, G97.52, M96.83, M96.830, M96.831, I97.62, I97.620, I97.621, J95.83, J95.830, J95.831, L76.2, L76.21, L76.22. Patients with preoperative sepsis and concurrent thoracic and/or lumbar procedures were excluded from analyses within our study.

Patients matching our inclusion criteria were systematically assessed for missing data. Variables with more than 25% of data missing were excluded from the analysis. For variables with 25% or less missing data, imputation was performed using the missForest package in the R statistical programming language (R Core Team, 2022) [15,16]. Population characteristics are summarized in Table 1. Among patients who developed postoperative hematoma requiring readmission and/or reoperation and those who did not, propensity score matching (PSM) was performed on a 1:5 basis using age and sex as matching variables. Propensity scores were calculated using the PsmPy package in the Python programming language [17,18]. This matching ratio was chosen to leverage the larger control group to improve precision and generalizability [19]. Matched population characteristics are summarized in Table 2.

**Table 1:** Full population characteristics (n = 54,427).

Characteristic	Mean (±SD) or percentage (n)
Age (y)	54.60 ± 11.52
Sex	
*Male*	50.21% (27,329)
*Female*	49.79% (27,098)
Race	
*Asian*	1.84% (1,003)
*African American or Black*	10.92% (5,942)
*Native American or Alaska Native*	0.69% (374)
*Caucasian or White*	86.25% (46,943)
*Native Hawaiian or Pacific Islander*	0.3% (165)
Hispanic ethnicity	5.03% (2,739)
Functional status	
*Partially or total dependence*	1.68% (912)
Body mass index (kg/m^2^)	30.45 ± 6.64
Setting	
*Inpatient*	69.79% (37,983)
*Outpatient*	30.21% (16,444)
ASA classification	
*1-No disturb*	3.61% (1,964)
*2-Mild disturb*	52.5% (28,575)
*3-Severe disturb*	41.7% (22,694)
*4-Life threat*	2.18% (1,188)
*5-Moribund*	0.01% (6)
Comorbidities	
*History of smoking*	27.35% (14,884)
*COPD*	4.55% (2,477)
*Diabetes*	16.28% (8,862)
*Chronic steroid use*	3.41% (1,857)
*Dyspnea*	5.02% (2,730)
*Hypertension*	45.4% (24,708)
*Bleeding disorder*	1.23% (669)
Preoperative lab values	
*Sodium*	139.44 ± 2.6
*BUN*	15.39 ± 6.22
*Creatinine*	0.91 ± 0.45
*WBC*	7.52 ± 2.46
*HCT*	41.62 ± 4.18
*Platelets*	248.53 ± 64.57
Preoperative transfusion	0.11% (58)
Procedure	
ACDF	
1 or 2 levels	78.07% (42,490)
≥3 levels	6.06% (3,298)
ACD w/o F	
1 or 2 levels	10.94% (5,952)
≥3 levels	0.65% (353)
Arthroplasty	
1 or 2 levels	2.86% (1,558)
≥3 levels	0.03% (17)
Corpectomy	
1 or 2 levels	9.41% (5,122)
≥levels	0.71% (387)
Posterior procedure	89.31% (48,610)
Operative time (min)	125.83 ± 65.12
Postoperative hematoma	0.32% (176)

**Table 2:** 5:1 Propensity matched population characteristics (n = 1,056).

Characteristic	Mean (±SD) or percentage (n)
Age (years)	58.23 ± 11.09
Sex	
*Male*	61.36% (648)
*Female*	38.64% (408)
Race	
*Asian*	1.14% (12)
*African American or Black*	11.36% (120)
*Native American or Alaska Native*	0.76% (8)
*Caucasian or White*	86.65% (915)
*Native Hawaiian or Pacific Islander*	0.09% (1)
Hispanic ethnicity	3.03% (32)
Functional status	
*Partially or total dependence*	1.14% (12)
Body mass index (kg/m^2^)	30.18 ± 6.0
Setting	
*Inpatient*	76.42% (807)
*Outpatient*	23.58% (249)
ASA classification	
*1-No disturb*	1.7% (18)
*2-Mild disturb*	52.18% (551)
*3-Severe disturb*	47.82% (464)
*4-Life threat*	2.18% (23)
*5-Moribund*	0.0% (0)
Comorbidities	
*History of smoking*	27.27% (288)
*COPD*	6.25% (66)
*Diabetes*	16.76% (177)
*Chronic steroid use*	3.03% (32)
*Dyspnea*	5.3% (56)
*Hypertension*	53.69% (567)
*Bleeding disorder*	1.42% (15)
Preoperative lab values	
*Sodium*	139.49 ± 2.69
*BUN*	15.94 ± 7.09
*Creatinine*	0.95 ± 0.59
*WBC*	7.54 ± 2.35
*HCT*	42.1 ± 4.8
*Platelets*	241.91 ± 65.38
Preoperative transfusion	0% (0)
Procedure	
ACDF	
1 or 2 levels	95.08% (1,004)
≥3 levels	2.08% (22)
ACD w/o F	
1 or 2 levels	4.64% (49)
≥3 levels	0.19% (2)
Arthroplasty	
1 or 2 levels	1.23% (13)
≥3 levels	0.0% (0)
Corpectomy	
1 or 2 levels	8.05% (85)
≥3 levels	0.47% (5)
Posterior procedure	95.27% (1,006)
Operative time (min)	143.3 ± 56.44
Postoperative hematoma	16.67% (176)

We employed PSM in our study to minimize confounding and selection bias inherent in retrospective observational data. Given the low incidence of postoperative hematoma requiring readmission or reoperation, previously cited at 0.4%, there was a significant imbalance between the number of cases and controls [2]. Without adjustment, comparisons between groups could be greatly skewed by the class imbalance in a small data set, rather than true associations with the outcome. PSM allows for the creation of a pseudo-randomized cohort by balancing key covariates between patients who developed postoperative hematoma and those who did not, thereby isolating the effect of clinical variables on hematoma formation. Prior studies have supported the use of one-to-many matching to improve precision and generalizability when dealing with rare outcomes [19]. Therefore, the use of PSM was critical to ensuring a robust foundation for our machine learning models while minimizing bias. However, it is important to note that shifting the event prevalence from 0.32% in the unmatched population to 16.67% in the matched cohort artificially inflates portions of algorithm’s predictive performance, Including positive predictive value (PPV) and accuracy. Nevertheless, due to the rarity of postoperative hematoma, model performance metrics were interpreted with particular attention to class imbalance and were not intended to support binary clinical decision-making, but rather to help identify variables associated with postoperative hematoma complication.

Six supervised ML classification algorithms were constructed and implemented in the Python programming language utilizing the SciKit-Learn library and the XGboost, LightGBM, and CatBoost frameworks [17,[20], [21], [22]]. The models included Random Forest Classifier (RF), Extreme Gradient Boosting Classifier (XGB), Light Gradient-Boosting Machine (LightGBM), Categorical Boosting (CatBoost), and Multilayer Perceptron Classifier (MLP). Additionally, a logistic regression model (LogReg) served as a baseline comparator to evaluate the relative performance and feature contributions of ML–based approaches. These algorithms were then tasked with stratifying the risk of postoperative hematoma formation requiring readmission and/or reoperation following anterior cervical spine surgery based on a given set of patient variables, including demographics, comorbidities, and preoperative lab values (see Table 2).

Initially, preprocessing of variables was completed using SciKit-Learn’s OneHotEncoder for categorical variables (eg, race, functional status) and SciKit-Learn’s Robust and Min-Max scalers for continuous variables (eg, age, BMI, lab values). Using Scikit-Learn’s train_test_split method, a 70:30 train: test split was performed, where 70% of our sample size was randomly divided into our training dataset, while the remaining 30% was set aside for final testing of the ML model’s performance. Each algorithm was trained using the Tree-Structured Parzen Estimator (TPE) algorithm for Bayesian optimization from the Opuntia library, combined with stratified 5-fold cross-validation to identify the optimal hyperparameters on the training data and ensure model generalizability. After determining the ideal hyperparameters, the final models were evaluated on the reserved testing data from the train-test split to assess their performance.

**Fig. 1:**
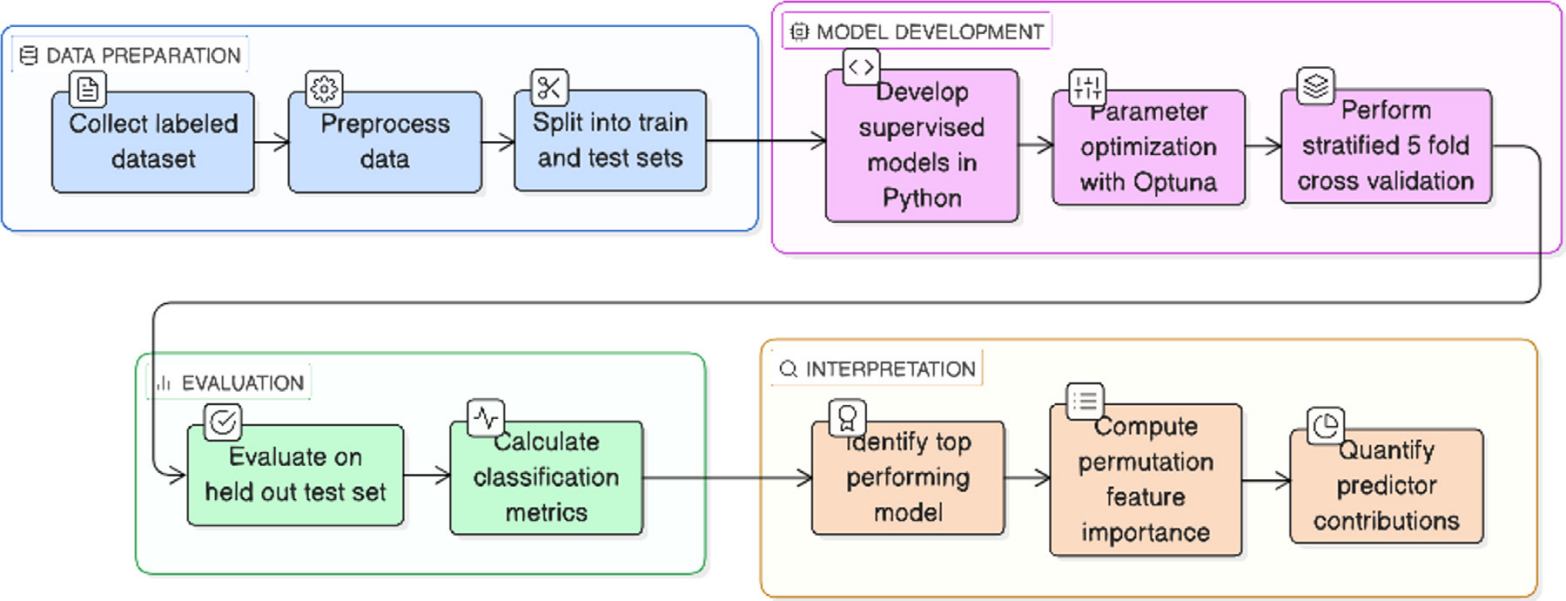
Schematic of machine learning model development, evaluation, and feature interpretation for prediction of postoperative hematoma following anterior cervical spine surgery.

The performance of the 6 ML models was evaluated using several commonly employed metrics, including classification accuracy, sensitivity, specificity, Positive Predictive Value (PPV), Negative Predictive Value (NPV), F1 score, Area Under the Receiver Operating Characteristics Curve (AUROC), and Area Under the Precision-Recall Curve (AUPRC). Notably, an F1 score is a machine learning metric used to evaluate models base on precision and sensitivity. An F1 score of 1.0 indicates perfect detection of all positive cases without generating any false positives or negatives. Graphical visualizations of the ROCs generated by the 3 models were created using the Matplotlib library in Python. The importance of each variable was assessed using permutation feature importance (PFI) with SciKit-Learn’s permutation importance function. PFI is calculated by measuring the change in model performance when a single variable is removed, disrupting the relationship between the feature and the predicted outcome. A higher PFI value indicates a greater impact on model performance when that feature is omitted. Since multiple ML algorithms were used in the study, the PFI values from the top-performing model were selected for further statistical analysis. Fig. 1 showcases an illustration of our methodology for model development, evaluation, and feature interpretation.

Finally, statistical analysis was performed using Python. Statistical significance was defined as p < .05. Differences between numerical variables were assessed using an independent sample t-test, while categorical variables were assessed using Pearson’s chi-square test. Continuous variables are presented as means and standard deviations (SD), while categorical variables are presented as frequencies in percentages.

## Results

Following a query of the ACS-NSQIP database and application of patient inclusionary factors, our search resulted in 54,427 patients undergoing anterior cervical spine surgery. Of the included patient sample, 176 (0.32%) patients sustained a postoperative hematoma requiring readmission and/or reoperation. Table 1 presents a detailed description of the demographics and population characteristics of the overall patient sample. 5:1 Propensity match was completed with details of the sample provided in Table 2.

**Fig. 2:**
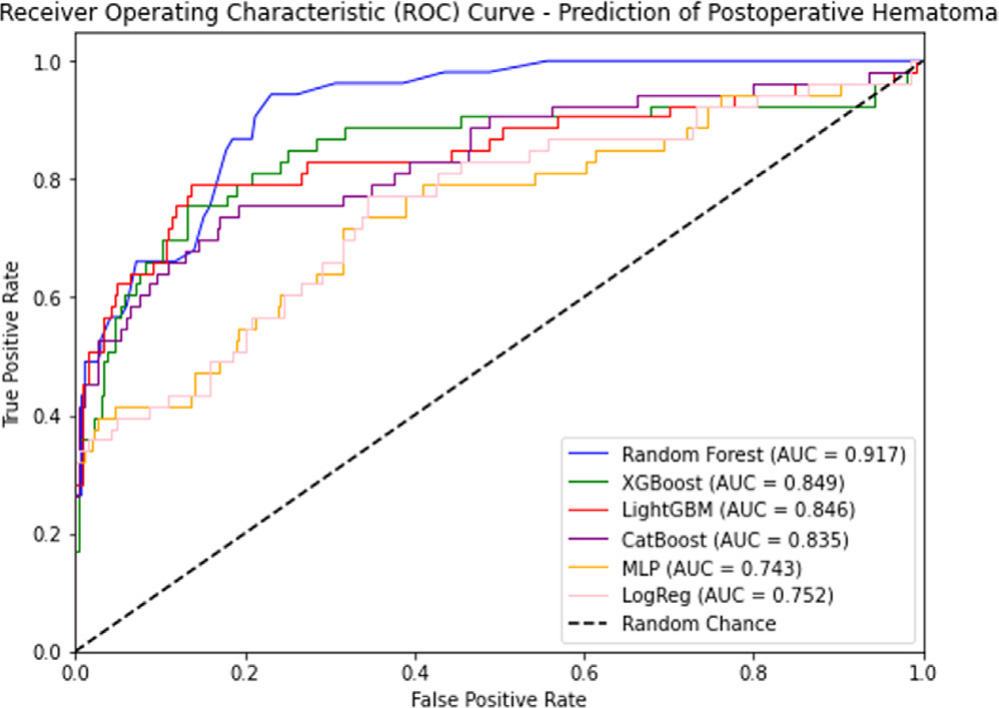
AUROC of machine learning algorithm-generated prediction of 30-day re-admission or re-operation for postoperative hematoma following anterior cervical spine surgery.

The 6 algorithms utilized in our analysis yielded an average accuracy of 87.75% as well as average sensitivity and specificity values of 30.2% and 99.3%, respectively. Regarding the predictive performance of the utilized algorithms, an average AUC value of 0.824 was observed along with an average F1 score of 0.451. Of the 6 algorithms, XGBoost algorithm performed best overall with AUC values of 0.849, a sensitivity of 35.9%, and the highest F1 score of 0.494 among the models, indicating the strongest balance between discrimination and case detection. Despite moderate discriminatory performance and high overall accuracy, the models demonstrated low sensitivity, reflecting an expected performance trade-off when attempting to predict an exceedingly rare outcome. The details of the receiver operating characteristics curve (ROC) generated by these algorithms can be reviewed in Fig. 2 and Table 4.

The average PFI (aPFI) values generated by the XGBoost algorithm were used to identify variables highly correlated with postoperative hematoma requiring readmission/reoperation. The variables identified in our analysis included the following: Diabetes (aPFI = 0.020, p = .020), preoperative WBC (aPFI = 0.028, p = .028), preoperative sodium (aPFI = 0.001, p = .001), and smoking history (aPFI = 0.026, p = .026). A record of the statistically significant and non-significant variables produced by this algorithm can be reviewed in Table 3. Unlike regression coefficients, which estimate the direction and magnitude of association under linear assumptions, permutation feature importance reflects a variable’s contribution to overall performance, allowing identification of clinically relevant features that may not meet traditional regression significance thresholds.

**Table 3:** Permutation feature importance for prediction of postoperative hematoma following anterior cervical spine surgery.

Outcome and features	Average importance (aPFI)	Mean ± SD or % sample (n) — Postoperative hematoma (n = 176)	Mean ± SD or % sample (n) — No postoperative hematoma (n = 880)	p
Operative time (min)	0.0369	136.15 ± 70.30	144.73 ± 53.17	.066
Diabetes	0.0043	22.7% (44)	15.6% (137)	**.020**
Preoperative BUN	0.0065	16.83 ± 12.63	15.76 ± 5.32	.070
Preoperative HCT	0.0032	41.80 ± 4.44	42.16 ± 4.87	.360
BMI	0.0020	29.56 ± 6.37	30.30 ± 5.92	.134
Preoperative WBC	0.0015	7.90 ± 2.53	7.47 ± 2.30	**.028**
Preoperative sodium	0.0008	138.89 ± 3.13	139.62 ± 2.58	**.001**
Dependent functional status	0.0007	4.0% (7)	0.6% (5)	**.001**
Preoperative creatinine	0.0005	0.95 ± 0.52	0.95 ± 0.60	.991
Smoking history	0.0003	34.1% (60)	25.9% (228)	**.026**

Bold values are statistically significant per *P*-value <0.05

**Table 4:** Performance of different predictive algorithms.

Algorithm predictive performance								
	Accuracy	Sensitivity	Specificity	PPV	NPV	F1	AUROC	AUPRC
Random forest	87.07%	0.2642	0.9924	0.8750	0.8704	0.4058	0.9173	0.7428
XGBoost	87.70%	0.3585	0.9811	0.7917	0.8840	0.4935	0.8487	0.6857
LightGBM	87.70%	0.2830	0.9962	0.9375	0.8738	0.4348	0.8461	0.7178
Catboost	87.38%	0.2642	0.9962	0.9333	0.8709	0.4118	0.8346	0.6875
Multilayer perceptron	88.01%	0.3208	0.9924	0.8947	0.8792	0.4722	0.7434	0.5569
Logistic regression	88.64%	0.3208	1.0	1.0	0.8800	0.4857	0.7525	0.5550
*Average*	***87.75%***	***0.3019***	***0.9931***	***0.9054***	***0.8764***	***0.4506***	***0.8238***	***0.6576***

PPV, positive predictive value; NPV, negative predictive value; F1, F1 score; AUROC, area under the receiver operating characteristic curve; AUPRC, area under the precision-recall curve.

## Discussion

As the population in the United States continues to age, the incidence of degenerative spine pathologies is expected to rise, with a subsequent increase in the prevalence of cervical spine surgery. Concurrently, with an expanding percentage of cervical spine surgeries being performed in outpatient ambulatory surgery centers or with a minimal period of inpatient monitoring, an emphasis has been placed on identifying risk factors that may predict undesired outcomes, such as unplanned readmission or reoperation [21,22]. Notably, a 5.4% readmission and 14% revision rate following anterior approach surgeries, such as ACDF, have previously been reported in the literature [23,24]. This study’s application of ML-based data analysis of the ACS-NSQIP dataset serves to identify a number of perioperative variables and patient characteristics that can allow for risk stratification of patients at risk for postoperative hematoma formation requiring either readmission or reoperation within the 30 days postoperatively. Notably, diabetes, a history of smoking, preoperative WBC, preoperative sodium, and dependent function status were identified as statistically significant variables that correlated and possibly contributing to this outcome. To our knowledge, this study is the first to apply machine learning methodologies to evaluate the clinical factors associated with readmission or reoperation due to postoperative hematoma formation following anterior cervical spine surgery.

Previous literature has aimed to evaluate the incidence and risk factors for postoperative hematoma following anterior cervical spine surgery using logistic regression. In a large ACS-NSQIP analysis of patients undergoing ACDF, Bovonratwet et al. identified lower BMI, preoperative INR > 1.2, male sex, ASA score > 3, and preoperative anemia as risk factors for postoperative hematoma formation [4]. Additionally, a previous single-institution study by O’Neill et al. identified idiopathic skeletal hyperostosis, the presence of ossification of the posterior longitudinal ligament, heparin use, longer operative time, and a greater number of vertebral levels as risk factors for postoperative retropharyngeal hematoma after anterior cervical spine surgery [13]. Ultimately, these studies cited an incidence rate for hematoma formation of 0.4% and 0.7%, respectively. Similarly, our study identified an incidence rate of 0.32%, highlighting both the rarity and clinical significance of this outcome. Differences in the specific variables identified across studies likely reflect differences in study design, patient populations, data granularity, and analytic methodology. Whereas prior investigations relied on regression-based models to estimate independent associations, the ML approaches used in the present study prioritize variables based on their contribution to overall risk stratification performance, allowing identification of nonlinear effects and interactions that may not be captured through conventional regression analyses.

ML models have proven to be reliable and efficient predictors of postoperative outcomes. For instance, Cabrera et al’s use of the ML RF algorithm to predict rate of reoperation following posterior cervical decompression in the presence of postoperative infection yielded an accuracy of 95.59%, a sensitivity of 43.33%, a specificity of 96.83%, and an AUC of 0.781 [9]. Similarly, Khan et. al’s use of ML to predict postoperative functional status following treatment of degenerative cervical myelopathy achieved an accuracy of 74.3%, sensitivity of 88.2%, specificity of 72%, and an AUC of 0.834 [25]. Notably, our study differs from prior investigations due to the significantly lower baseline incidence of the primary outcome. The average ML model employed in our study identified risk factors associated with postoperative hematoma requiring readmission and reoperation with an accuracy of 87.75%, sensitivity of 30.19%, specificity of 99.31%, and an AUC of 0.7823. This performance profile reflects the challenges of the accuracy paradox common in highly imbalanced datasets, where a predictive model could achieve a high baseline accuracy simply by predicting the absence of a very rare event for all patients. The observed combination of moderate AUROC, high accuracy, and low sensitivity represents a common trade-off in rare-event prediction, wherein models preferentially optimize discrimination while accepting reduced case detection. As a result of this trade-off, these models are better suited for risk stratification rather than for screening or rule-out applications.

The use of predictive algorithms to identify the risk factors described in this study may allow surgeons to more effectively identify surgical candidates who require heightened perioperative vigilance. While these models cannot definitively dictate outpatient versus inpatient surgical setting due to their low case-detection rate, patients presenting with multiple identified high-risk features may be better managed with a prolonged period of inpatient monitoring or may benefit from targeted intervention to decrease their risk of hematoma formation before an outpatient procedure is attempted. Additionally, understanding the risk variables of this rare occurrence can assist providers in deciding which patients warrant more frequent evaluations in the postoperative period. The novelty of this study lies in the application of ML classifiers alongside a regression baseline to characterize postoperative hematoma risk, thereby extending prior regression-based findings.

Crucially, because preoperative machine learning models possess a low sensitivity (30.2%) and cannot reliably rule out the development of an individual postoperative hematoma, they represent only an initial step in risk mitigation. The definitive line of defense against catastrophic respiratory compromise remains the implementation of robust postoperative recognition and treatment systems. This is particularly vital in the contemporary landscape of expanding outpatient and short-stay anterior cervical procedures, where rapid clinical deterioration can occur away from standard inpatient floor checks. Preoperative stratification serves exclusively to amplify postoperative vigilance. Ultimately, in cases of rare complications, predictive algorithms cannot substitute for real-time clinical intuition or the necessity for a rapidly responsive care environment.

### Limitations

This study is not without its limitations. One limitation inherent to our topic of interest is the rarity of postoperative hematoma formation requiring readmission and reoperation. The low incidence of this outcome produces an imbalance in the datasets utilized by our study, which, in turn, may skew the overall data sample and risk factors discussed within this study. Propensity matching was utilized to combat class imbalance. While this approach improves internal validity for a rare outcome, it limits the generalizability of absolute risk estimates. Most importantly, the extremely low base rate (0.32%) of hematoma formation creates an aggressive mathematical trade-off, thereby skewing the performance metric of machine learning algorithms before and after propensity matching. Therefore, model outputs should be interpreted strictly as supporting risk awareness rather than an independent clinical decision-making tool. Furthermore, as with any insurance-based national database, errors in coding may occur, resulting in under- or over-reporting of the true number of anterior cervical spine cases, as well as the incidence of postoperative hematoma. It is also important to note, the ACS-NSQIP database includes data from hospitals that have the infrastructure to collect and organize data, specifically 707 United States hospitals, thus possibly limiting its generalizability. Finally, the database is limited to only 30 days postoperatively; complications related to a postoperative hematoma, while exceedingly rare at such a far time-point after surgery, were not included in the analysis. Additionally, a notable limitation of this study is the current lack of external or temporal validation. While internal consistency and performance were rigorously evaluated using internal train-test splitting and cross validation techniques within the database, the algorithms have not been evaluated on distinct, independent patient population. Therefore, external validation remains essential to ensure generalizability of the associated risk factors identified within these models. Lastly, while PFI can be used to highlight a variable’s potential clinical relevance, it does not indicate the intrinsic significance of any specific risk factor. Instead, PFI measures the change in a model’s performance when a particular variable is removed or randomized within a dataset, offering an indirect assessment of the feature’s contribution to accurate outcome predictions. While ML models demonstrated improved discrimination compared with regression, they do not obviate the interpretability and inferential strengths of traditional regression analyses, and both approaches should be viewed as complementary. Ultimately, while ML algorithms offer valuable insights into key predictors of outcomes, they cannot replace a provider's clinical judgment or the holistic approach to patient care.

## Conclusion

Application of MLAs to the ACS-NSQIP database identified a number of clinical variables that were deemed predictive of postoperative hematoma formation following anterior cervical spine surgery. As such, these findings may serve to help clinicians mitigate this rare complication. Additionally, these findings further expand the clinical relevance of such factors by providing quantification of the relative contribution of each variable to producing postoperative hematoma. This information provides clinicians with internally validated and quantified predictors of postoperative hematoma that may serve to guide perioperative decision-making in this high-risk and medically complex patient population.

## Funding

The authors received no financial or material support for the research, authorship, and/or publication of this article.

## Ethical approval

Informed consent was not required for this study due to the nature of this study. This decision was made by the Loma Linda University Health Institutional Review Board (IRB) which deemed the project exempt.

## Data availability statement

The data that support the findings of this study are not publicly available due to information that could compromise the privacy of research participants but are available upon reasonable request.

## Declaration of competing interests

The authors declare that they have no known competing financial interests or personal relationships that could have appeared to influence the work reported in this paper.
